# Histone H3K36me2 demethylase KDM2A promotes bladder cancer progression through epigenetically silencing RARRES3

**DOI:** 10.1038/s41419-022-04983-7

**Published:** 2022-06-13

**Authors:** Bing Lu, Jiatian Wei, Houhong Zhou, Jie Chen, Yuqing Li, Liefu Ye, Wei Zhao, Song Wu

**Affiliations:** 1 Institute of Urology of Shenzhen University, The Third Affiliated Hospital of Shenzhen University, Shenzhen Luohu Hospital Group, Shenzhen, 518000 China; 2 Key Laboratory of Stem Cells and Tissue Engineering (Sun Yat-sen University), Ministry of Education, Guangzhou, 510080 China; 3 Sun Yat-sen Memorial Hospital, Sun Yat-sen University, Guangzhou, 510080 China; 4 Department of Urology, Fujian Provincial Hospital, 134 Dong Street, Fuzhou, 350001 China

**Keywords:** Urological cancer, Cell growth

## Abstract

Epigenetic dysregulation contributes to bladder cancer tumorigenesis. H3K36me2 demethylase KDM2A functions as an important epigenetic regulator of cell fate in many types of tumors. However, its role in bladder cancer remains unknown. Here, we revealed a positive correlation between *KDM2A* gene copy number gain and upregulation of *KDM2A* mRNA expression in bladder cancer. Moreover, a super-enhancer (SE) driving *KDM2A* transcription was found in high-grade bladder cancer, resulting in a significantly higher expression of *KDM2A* mRNA compared to that in low-grade bladder tumors. *KDM2A* knockdown (KD) decreased the proliferation, invasion, and spheroid formation of high-grade bladder cancer cells and inhibited tumor growth in mouse xenograft models. Furthermore, we identified *RARRES3* as a key KDM2A target gene. KDM2A suppresses *RARRES3* expression via demethylation of H3K36me2 in the *RARRES3* promoter. Intriguingly, *RARRES3* KD attenuated the inhibitory effects of KDM2A depletion on the malignant phenotypes of high-grade bladder cancer cells. The combination of the KDM2A inhibitor IOX1 and the RARRES3 agonist all-trans retinoic acid (ATRA) synergistically inhibited the proliferation of high-grade bladder cancer cells, suggesting that the KDM2A/RARRES3 axis may be a promising therapeutic target for the treatment of high-grade bladder cancer.

## Introduction

Until recently, little progress had been made in the treatment of bladder cancer. Unfortunately, the five year survival rate has remained unchanged for 30 years [1]. Approximately 75% of newly diagnosed bladder cancer patients have non-muscle-invasive bladder cancer (NMIBC), including stage Ta, stage T1, and carcinoma in situ (CIS) [2]. High-grade bladder cancer is an extremely aggressive malignancy that is associated with high morbidity and mortality rates [3]. Approximately 69–80% of high-grade patients relapse and have a 33–48% chance of progression to muscle-invasive bladder cancer (MIBC) [4, 5]. Understanding the molecular features of high-grade bladder cancer may improve biomarkers for risk stratification and treatment effectiveness.

The deregulation of histone modifications has emerged as an important mechanism in bladder carcinogenesis. Whole-exome sequencing demonstrated that the mutation frequency of chromatin remodeling genes in bladder cancer was more than 10%, including *KDM6A*, *MLL2* (also known as *KMT2D*), *CREBBP*, *EP300*, and *ARID1A* [6]. KDM6A acts as a tumor suppressor by demethylating histone H3 lysine 27 (H3K27) and opening chromatin configuration [7]. Mutation of *KDM6A* confers bladder tumors with certain competitive advantages that drive cancer cells to colonize larger regions of the urothelium [8]. A recent study showed that *MLL2*, which encodes a histone H3 lysine 4 (H3K4) methyltransferase, was amplified at a rate of 27% in bladder cancer [6]. Moreover, mutations in *MLL2* and *KDM6A* were mutually exclusive, indicating that mutations in the two genes have redundant downstream effects on carcinogenesis, or that the combined loss is synthetically lethal [9]. Nonetheless, predicting the progression of high-grade bladder cancer via histone modifier mutations remains a challenge.

Lysine demethylase 2 A (KDM2A), also known as FBXL11 and JHDM1A, is highly expressed in ovarian [10], pancreatic [11], colorectal [12], breast [13], and gastric cancer [14]. As a Jumonji-C domain-containing histone demethylase, KDM2A is associated with inactively transcribed genes by demethylating the dimethylated H3K36 (H3K36me2) residue; however, it exerts little or no activity on monomethylated and trimethylated H3K36 residues [15]. H3K36me2 is enriched in both intergenic regions and gene bodies and plays an intriguing epigenetic regulatory role in cell proliferation [16], differentiation [17, 18], and apoptosis [19]. Abnormal KDM2A expression is associated with tumorigenesis [20], metastasis [21], chemotherapy resistance [22], and poor prognosis [20]. Studies have demonstrated that mild glucose starvation promotes KDM2A‑induced demethylation of H3K36me2, which decreases rRNA transcription and proliferation via the AMPK signaling pathway in breast cancer [13]. KDM2A reverses epithelial-to-mesenchymal transition (EMT) by regulating the PI3K signaling pathway to promote ovarian cancer progression [10]. However, the function of KDM2A in bladder cancer remains largely unknown.

Here, we report a positive correlation between the increase in *KDM2A* expression and gain in gene copy number in bladder cancer. H3K27ac ChIP-seq analysis revealed a specific super-enhancer (SE) at the *KDM2A* locus in high-grade bladder cancer. Moreover, we included the data of *KDM2A* knockdown (KD) on proliferation, invasion, spheroid formation, tumorigenesis, and mRNA expression to characterize the functions of KDM2A in high-grade bladder cancer. This study identified several key targets of KDM2A, including *RARRES3*, which has not been previously reported in high-grade bladder cancer. The study also identified the KDM2A/RARRES3 axis as a potential therapeutic target for the treatment of high-grade bladder cancer.

## Materials and methods

### Cell culture

Human bladder cancer line UMUC3 was cultured in Dulbecco’s modified Eagle’s medium (DMEM, Gibco, Cat. No.12430104), 5637, RT4 and MGHU3 were cultured in RPMI-1640 medium (Gibco, Cat. No.22400105). All media were supplemented with 10% fetal bovine serum (FBS, Biological Industries) and 100 U/mL penicillin/streptomycin (Gibco, Cat. No.15140-122). Cells were grown at 37 °C with 5% CO_2_. Patient specimens were collected from the Department of Urological Surgery at the Affiliated Luohu Hospital of Shenzhen University. Specimens were examined by a pathologist to verify tumor types and grades. All procedures in the patients were approved by the ethics committee of the Affiliated Luohu Hospital of Shenzhen University.

### Constructs of shRNA and cell transfection

The shRNA sequences against target genes were designed and cloned into the pLKO‐puro lentiviral vector. The sequence of KDM2A shRNA (shKDM2A-1: 5′-ATGCCACGCTTC GCCTCATAA-3′; shKDM2A-2: 5′-GCTTACTCCACCGGCTGATAA-3′) and sequence of RARRES3 shRNA (5′- CACGGCAAGACACCTGTAGA -3′) were synthesized by Sangon Biotech (Shanghai, China) and cloned into the lentiviral vector pLKO‐puro (Addgene, 8453). To generate lentiviral particles, the constructed shRNA expression plasmid was co‐transfected with packaging plasmids pVSVG and psPAX2 into human embryonic kidney 293 T cells using lipofectamine 2000 (Invitrogen, Cat. No.11668019). After 48 h, the viral supernatant was collected. UMUC3 and 5637 cells were infected with the obtained lentiviruses. Then, the infected cells were treated with the medium containing 2 mg/L puromycin (Solarbio, Cat. No. P8230) for 3 day.

### Animal studies

Male BALB/c nude mice (18 ± 2 g, 4–6 weeks) were purchased from the Model Animal Research Center of Nanjing University (China). All animal experiments were conducted under the guidance of the Ethical Committee of The Affiliated Luohu Hospital of Shenzhen University. The mice were randomly divided into 3 groups and subcutaneously injected with ShC, shKDM2A-1 and shKDM2A-2 UMUC3 cells (1 × 10^7^ cells per mouse, and 6 mice per group). As for metastasis model, cells were injected into the nude mice via tail vein (2 × 10^6^ cells per mouse, and 6 mice per group). Tumor growth was monitored every 3 days by measuring the width (W) and length (L) with calipers. The volume (V) of the tumor was calculated using the formula V = (W^2^ × L) / 2.

### Cell proliferation assay

Cell proliferation ability was evaluated using the CellTiter-Glo ^®^ Luminescent Cell Viability Assay according to the manufacturer’s instructions. In short, the transfected bladder cancer cells were seeded in 96-well plates (Fisher Scientific) at 3000 cells/well densities. The cell numbers were measured at 0, 12, 24, 36, 48, 60, and 72 h, respectively.

### Assessing drug synergism

IOX1 (Cat. No. HY-12304) and all-trans-Retinoic acid (ATRA, Cat. No. HY-14649) were purchased from MedChemExpress (MCE). To test the combinatory effects of IOX1 and ATRA, the cells were seeded into 96-well plates and then subjected to the drug at indicated concentration for 48 h. Combination index (CI) was calculated by CalcuSyn software (Biosoft, Cambridge, UK). CI less than 1.0 is considered as synergistic effect.

### Transwell assay

Cell invasion assays were performed in 24-well Transwell cell culture chambers pre-coated with matrigel basement membrane gel (Corning, Cat. No. 354234). DMEM containing 10% fetal bovine serum (FBS) were added to the lower chambers. Meanwhile, 50,000 cells were seeded into the upper chamber with 300 μl culture medium without serum. After incubation at 37 °C for 24 h, the cells in upper chamber was fixed with 4% paraformaldehyde for 10 min and stained with 0.1% crystal violet for another 20 min at room temperature. The invading cells were observed and counted under an optical microscope (magnification, ×100).

### Total RNA extraction and quantitative real-time PCR (qPCR)

Total RNA was isolated from cells using TRIzol reagent (Cat. No. 15596026; Thermo Fisher Scientific, Inc.) and quantified using NanoDrop 2000 according to the manufacturer’s protocol. HiScript II Q^RT^ SuperMix kits (Cat. No. R223-01, Vazyme) were used for the reverse-transcription reactions. Primers for qPCR were as follows: *KDM2A*, forward primer (5’-CAACAGC GATCCCAAGTTAGC-3’) and reverse primer (5’-TGGCCGAGTGGGGAATTTAAG-3’); *RARRES3*, forward primer (5’-GAGCAGGAACTG TGAGCACT-3’) and reverse primer (5’- TTGGCCTTTTCC ACCTGTTT-3’) and *GAPDH*, forward primer (5’- ACCA-CAGTCCATG CCATCAC −3’) and reverse primer (5’- CCACCACCCTGTTGCTG −3’). qPCR was carried out using ChamQ Universal SYBR qPCR Master Mix (Cat. No. Q711-02, Vazyme) on a Life Quant Studio 6 Flex Real-time PCR system.

### Apoptosis and cell cycle assay

For cell cycle analysis, UMUC3 and 5637 cells were collected and fixed with 3 mL 70% ethanol at −20 °C. The cells were stained with 1 mL cell cycle staining solution (MULTI SCIENCES, Cat. No.70-CCS012) for 30 min on the ice in the dark. The fluorescence intensities of propidium iodide (PI) were recorded using a flow cytometer. For cell apoptosis analysis, cells were incubated with annexin V-APC and 7-AAD (Annexin V-APC/7-AAD apoptosis kit, MULTI SCIENCES, Cat. No.70-AP105-100). Flow cytometry assay was performed using BD LSRFortessa (BD Biosciences, CA, USA).

### Spheroid formation assay

The cells were digested into single-cell suspensions. 1000 cells were plated into ultra-low attachment 96-well plates (Corning) in DMEM/F12 medium with 10 ng/ml mL Epidermal Growth Factor (EGF, PeproTech, Cat. No. AF-100-15-500),10 ng/ml mL basic fibroblast growth factor (bFGF, PeproTech, Cat. No. AF-100-18B-100) and B27 supplement (Invitrogen, Cat. No.17504-044) for 7 days.

### RNA-seq and data analysis

RNA-seq was performed as previously descripted. Trim_galore and Fastqc were used for filtering raw sequencing reads [23]. Sequencing reads were aligned to the UCSC hg19 human genome reference via hisat2. High quality and uniquely aligned reads were counted at gene regions using the package Gfold based on Gencode Human GRCh37 annotations. Differential gene expression analysis between different groups was performed using the R/Bioconductor package DESeq with contrast adjustment for multiple groups comparison. Gene ontology (GO) and Kyoto Encyclopedia of Genes and Genomes (KEGG) pathway enrichment analysis were done using R/Bioconductor package clusterProfiler and cytoscape. Heatmaps were drawn using the R package Pheatmap and ggplot2.

### Chromatin immunoprecipitation (ChIP)-seq and ChIP-qPCR

ChIP using 2 × 10^6^ to 10 × 10^6^ cross-linked cells were performed as previously descripted [24]. The following antibodies were used for ChIP: rabbit anti-KDM2A (Novus Biologicals, Cat. No. NB100-74602), rabbit anti-H3K36me2 (Abcam, Cat. No. ab176921) and Rabbit IgG (Abcam,Cat. No. ab172730). The primers used for ChIP-qPCR are as follow: *RARRES3*-1, forward primer (5’-GGGGCCCCCTTAAAGAGTTT-3’) and reverse primer (5’-TTCACTGTGGGCTGAGTCAC -3’), *RARRES3*-2, forward primer (5’-CACGGCAAGACACCTGTAGA-3’) and reverse primer (5’- GATGTTGCTGTGTTTGGGCA -3’), *RARRES3*-3, forward primer (5’-AAAATGGGCCACTCAAGGCT-3’) and reverse primer (5’-TGTCTCCATTGCACTGGTCC -3’).

### Statistical analysis

All experiments were repeated at least three times and the data were expressed as mean ± standard deviation. Two-tailed Student’s t-tests were used for comparisons between the two groups. One-way analysis of variance (ANOVA) with Bonferroni’s test was used for multiple comparisons. All the charts were drawn by GraphPad Prism 7.0 (GraphPad Software Inc.). Differences were considered statistically significant at two-tailed *P* < 0.05 (*P < 0.05, **P < 0.01, ***P < 0.001).

## Results

### KDM2A gene copy number gain contributes to KDM2A mRNA up-regulation in bladder cancer

**Fig. 1:**
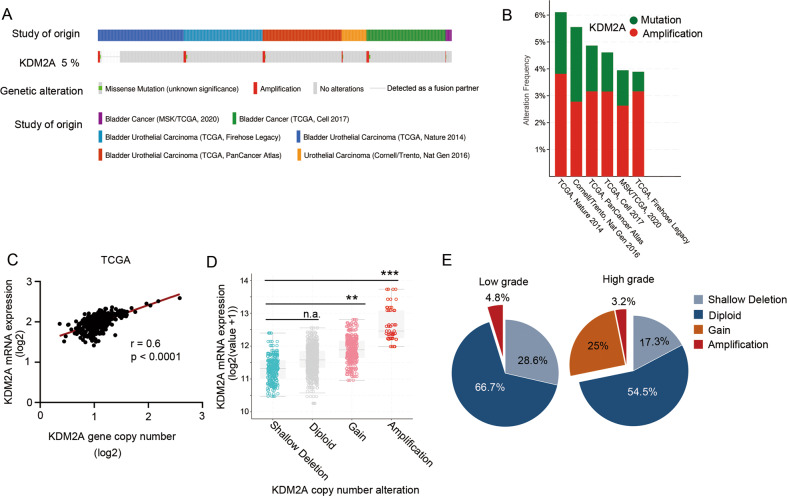
*KDM2A* gene copy number gain contributed to *KDM2A* mRNA upregulation in bladder cancer. **A** The OncoPrint tab summarizes KDM2A genomic alterations in six sample sets. Each column represents a tumor sample, red bars indicate gene amplifications, blue bars are homozygous deletions, and green squares are missense mutations (http://www.cbioportal.org/). **B** Further analysis of KDM2A alterations in each of the studies. **C** *KDM2A* mRNA expression was positively correlated with *KDM2A* gene copy number alteration (r = 0.6, *P* < 0.0001, linear regression). **D** *KDM2A* gene copy number gain and amplification were positively correlated with increased mRNA expression in TCGA bladder cancer cohort. **E** TCGA gene copy number data showed a higher ratio of gain and amplification alterations detected in high-grade bladder cancer than in low-grade samples (high-grade, 28.2%; low-grade, 4.8%). *P < 0.05; **P < 0.01; ***P < 0.001 is based on the Student’s *t* test. All results are from more than three independent experiments. Values are mean ± SD.

We analyzed genetic variations in *KDM2A* in 1916 bladder cancer cases retrieved from six studies (476 cases from Memorial Sloan Kettering [MSK]/The Cancer Gene Atlas [TCGA], 2020; 413 cases from TCGA, Cell 2017; 131 cases from TCGA, Nature 2014; 413 cases from TCGA, Firehose Legacy; 411 cases from TCGA, PanCancer Atlas; and 72 cases from Cornell/Trento, Nat Gen 2016). A total of 5% of bladder cancer patients have *KDM2A* mutations which are frequently amplified (Fig. 1A, B). In addition, there was a positive correlation between *KDM2A* mRNA expression and *KDM2A* gene copy number gain in patients with bladder cancer (Fig. 1C). Notably, the expression level of *KDM2A* in patients with amplification and gain was significantly higher than that in patients with shallow deletion and diploid (Fig. 1D). Additionally, we analyzed TCGA bladder cancer datasets and found that the proportion of *KDM2A* amplification and gain alteration in high-grade bladder cancer was significantly higher than that in low-grade bladder cancer (Fig. 1E).

### High expression level of KDM2A in high-grade bladder cancer is due to SE formation

**Fig. 2:**
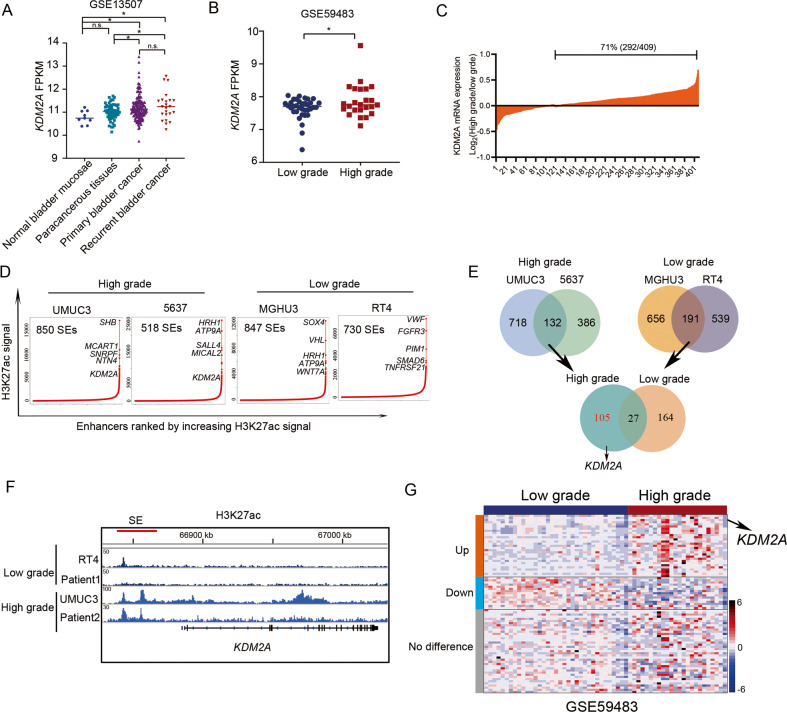
High-expression level of *KDM2A* was related with H3K27ac enrichment in high-grade bladder cancer. **A** Analysis of *KDM2A* expression level in bladder cancer tissues and normal bladder tissues in GEO database (GSE13507). **B** Analysis of *KDM2A* expression level in high-grade and low-grade bladder cancer in GEO database (GSE59483). **C** Compared with low-grade bladder cancer, 71% of patients with high-grade bladder cancer had upregulated expression of *KDM2A* in *TCGA*. **D** Enhancers were ranked by increasing H3K27ac signal in high-grade (UMUC3, 5637) and low-grade (MGHU3, RT4) bladder cancer cells. The population above the inflection point of the curve are defined as SEs. The number of SEs and examples of genes associated with SEs are shown for each sample. **E** Venn diagrams depicting the integrative analysis of SE-associated genes between the high-grade and low-grade bladder cancer cells. **F** H3K27ac ChIP-seq peaks at the *KDM2A* loci in bladder cancer cells and samples. **G** Heatmap depicts the expression of 105 SE-associated genes specific to high-grade in bladder cancer samples (GSE59483). *P < 0.05; **P < 0.01; ***P < 0.001 is based on the Student’s *t* test. All results are from more than three independent experiments. Values are mean ± SD.

Next, we found that the expression of *KDM2A* in primary and recurrent bladder cancer was significantly higher than that in paracancerous tissues (Fig. 2A). Intriguingly, high-grade bladder cancer showed higher *KDM2A* expression than low-grade bladder cancer (Fig. 2B, C).

Emerging evidence indicates that cancer cells hijack SEs to drive oncogenic expression [25]. To identify SEs in bladder cancer, we performed H3K27ac ChIP-seq assay and ranked all putative SEs by H3K27ac enrichment in low-grade (MGHU3 and RT4) and high-grade bladder cancer cell lines (UMUC3 and 5637) (Fig. 2D). Although SEs were heterogeneous among bladder cancer cell lines, we identified 105 high-grade and 164 low-grade bladder cancer-specific SEs. Importantly, the *KDM2A* locus is found driven the SE in both high-grade bladder cancer cell lines (Fig. 2E). Moreover, we confirmed the formation of SE at the *KDM2A* locus in high-grade bladder cancer in surgical specimens from patients with bladder cancer (Fig. 2F). Furthermore, the expression of 105 high-grade specific SE-driven genes was ranked, and *KDM2A* was found to be the most significantly upregulated (Fig. 2G). These findings indicate that DNA mutations may mediate oncogenic SE formation, triggering *KDM2A* expression and bladder cancer progression.

### KDM2A is essential for malignant phenotypes of high-grade bladder cancer cells

**Fig. 3:**
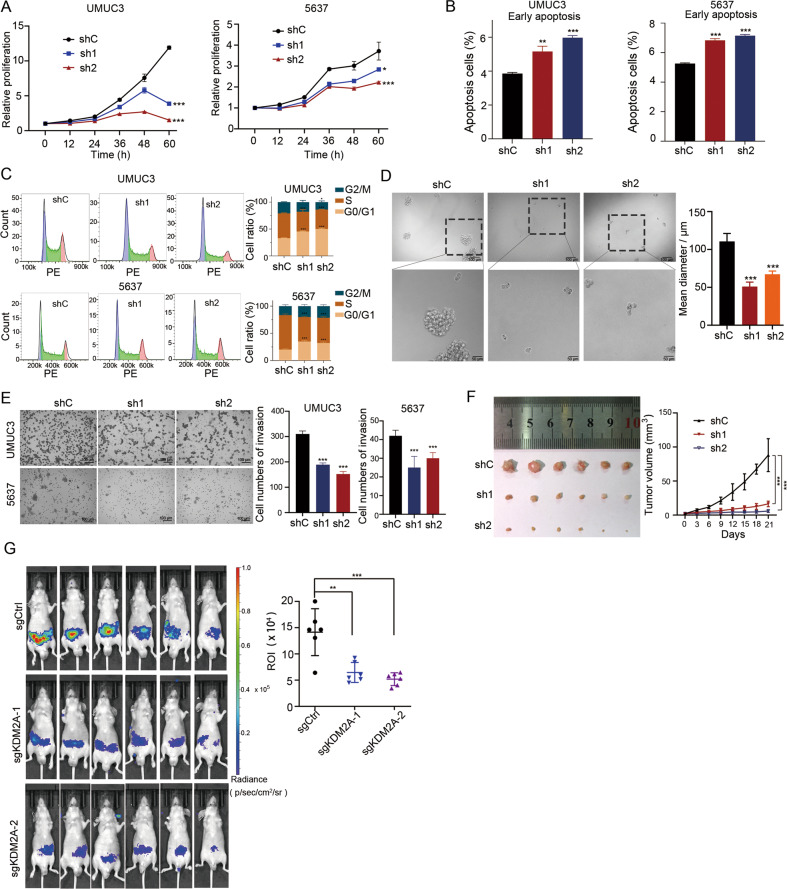
KDM2A KD suppressed bladder cancer cell proliferation and migration. **A** Cell viability assays to investigate the proliferation ability of UMUC3 and 5637 cells that stably transduced with non-targeting scrambled control shRNA (shCtrl) or two KDM2A shRNAs (shKDM2A-1 and shKDM2A-2). **B**, **C** Flow cytometry analysis of early apoptosis staining by annexin V and PI and cell cycle staining by PI in UMUC3 and 5637 cells upon KDM2A KD. **D** Sphere-forming assay was performed on UMUC3 cells following KDM2A KD. The sphere size was measured on day seven. **E** Invasion assay was performed to assess UMUC3 and 5637 cells upon KDM2A KD with two different shRNA targets. Cell invasion was assessed by counting the number of migrating cells after 24 h. **F** KDM2A KD in UMUC3 cells were subcutaneously injected into BALB/c nude mice (*n* = 6 per group). Left: images of tumor tissue in the control and KDM2A KD groups (day 21). Right: the volumes of xenograft tumors of nude mice derived from subcutaneous implantation of UMUC3 cell lines. **G** Bioluminescence in vivo imaging showing the metastases of nude mice (*n* = 6 per group) injected (i.v.) with *KDM2A* KO or control UMUC3 cells. *P < 0.05; **P < 0.01; ***P < 0.001 is based on the Student’s *t* test. All results are from more than three independent experiments. Values are mean ± SD.

To investigate the effects of KDM2A depletion in high-grade bladder cancer cells, we stably silenced *KDM2A* in high-grade bladder cancer cell lines (UMUC3 and 5637) using two specific shRNAs (Supplementary Fig. 1A, B). *KDM2A* KD significantly inhibited the proliferation of both UMUC3 and 5637 cells (Fig. 3A). It also resulted in an increase in early apoptotic cells and G0/G1 phase cell cycle arrest in these cell types (Fig. 3B, C).

To test the effects of *KDM2A* depletion on the malignant phenotypes of high-grade bladder cancer cells, spheroid formation and invasion assays were performed in *KDM2A* KD and control UMUC3 and 5637 cells. Cells expressing sh*KDM2A* showed a significantly reduced sphere-forming propensity (Fig. 3D) and invasive activity (Fig. 3E). Moreover, we demonstrated that depletion of *KDM2A* resulted in a significant decrease in tumor size compared to that in the vehicle control (Fig. 3F). We also constructed *KDM2A* knockout (KO) UMUC3 cell lines (Supplementary Fig. 1C), and examined the metastatic capacity of *KDM2A* KO UMUC3 cells in the mice model. We found that KO of *KDM2A* significantly suppressed the metastasis of UMUC3 cells in vivo compared with sgControl (Fig. 3G). In addition, KO of *KDM2A* increased the expression of MET markers, including *CDH1* and *SIM2*, but decreased the expression of EMT marker genes (Supplementary Fig. 1D). Taken together, these results suggested that *KDM2A* KD significantly reduced high-grade bladder cancer cell proliferation and metastasis.

### KDM2A binds and modulates H3K36me2 at the *RARRES3* locus

**Fig. 4:**
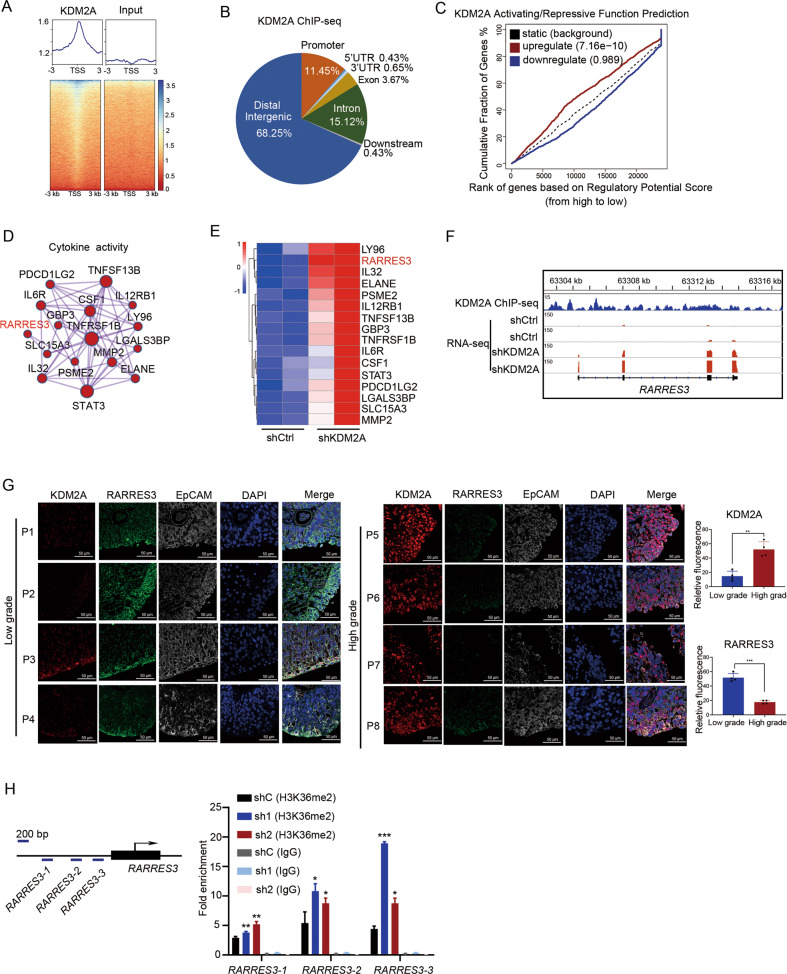
KDM2A binds and modulates tumor suppressor RARRES3. **A** Heatmap showed occupancy for KDM2A across the genome in UMUC3 cells. **B** Genome-wide distribution of KDM2A ChIP-seq peaks in UMUC3 cells. **C** Cumulative distribution curve shows KDM2A activating or repressing effects. Differential genes obtained from RNA-seq after KDM2A KD were divided into three groups: upregulated, downregulated, and unchanged according to the expression pattern. The corresponding genes of KDM2A ChIP-seq were sorted from high to low according to regulatory potential score, and the cumulative distribution curves of upregulated, downregulated, and invariant genes were made. **D** GO terms of upregulated genes that KDM2A target directly. **E** Heatmap showed the ranking of upregulated cytokine activity genes upon KDM2A KD. **F** Genome browser tracks of KDM2A ChIP-seq data and RNA-seq data in RARRES3 gene loci. **G** The protein level of KDM2A, RARRES3, and EpCAM (the biomarker of bladder cancer) by immunofluorescence staining assays. **H** ChIP-qPCR showed the H3K36me2 modification of *RARRES3* promoter upon KDM2A KD. *P < 0.05; **P < 0.01; ***P < 0.001 is based on the Student’s *t* test. All results are from more than three independent experiments. Values are mean ± SD.

To identify the genes directly controlled by KDM2A, we performed KDM2A ChIP-seq experiments and sought to characterize the genome-wide occupancy of KDM2A (Fig. 4A). Many ChIP-seq peaks were located at the distal intergenic (68.25%), intron (15.12%), and promoter (11.45%) regions (Fig. 4B). Concurrently, we analyzed genes whose expression levels changed significantly after KDM2A depletion (Supplementary Fig. 2A). Following gene ontology (GO) analyses, these upregulated genes were found to be enriched in the degradation of the extracellular matrix (ECM) (Supplementary Fig. 2B), and downregulated genes were found to be enriched in wound healing and DNA replication (Supplementary Fig. 2C).

To characterize the regulatory potential imposed by KDM2A, we sought to integrate KDM2A binding loci and RNA-seq data of *KDM2A* KD and control UMUC3 cells. From the KDM2A activating/repressive function prediction results, the upregulated KDM2A binding genes had a higher regulatory potential score than the downregulated and unregulated genes (Fig. 4C). Interestingly, depletion of *KDM2A* affected the expression of KDM2A binding genes associated with GO terms linked to cytokine activity (Fig. 4D) and extracellular matrix structural constituents (Supplementary Fig. 2D). Ranking the change multiples of the significantly upregulated cytokine activity genes (Fig. 4E), we identified the tumor suppressor retinoic acid receptor responder 3 (*RARRES3*) as one of the critical targets of KDM2A (Fig. 4F). Importantly, *RARRES3* expression negatively correlated with the mRNA expression of *KDM2A* in bladder cancer (Supplementary Fig. 2E). In addition, we collected the tumor tissues from 4 cases of high-grade and 4 cases of low-grade bladder cancer patients (Supplementary Fig. 2F). The protein level of KDM2A in high-grade bladder cancer was higher than that in low-grade bladder cancer. Contrastingly, the protein level of RARRES3 in high-grade bladder cancer was significantly lower than that in low-grade bladder cancer (Fig. 4G).

KDM2A plays an intriguing epigenetic regulatory role and catalyzes the demethylation of H3K36me2 [15]. Thus, we performed H3K36me2 ChIP-qPCR to characterize H3K36me2 modification at the *RARRES3* promoter in KDM2A KD and control UMUC3 cells. *KDM2A* silencing significantly increased H3K36me2 modification at the *RARRES3* locus in high-grade bladder cancer cells (Fig. 4H). The KDM2A ChIP-qPCR assays results showed the enrichment of KDM2A at promoters of the target genes, suggesting the recruitment of KDM2A on these genomic sites (Supplementary Fig. 2G). Moreover, four transcriptional factors (EP300, FOS, FOSL1, and JUN) were predicated as *RARRES3* transcriptional regulator (https://meme-suite.org/meme/tools/fimo). ChIP-qPCR assays showed that KD of *KDM2A* increased the enrichment of the four transcriptional regulators on the promotor of *RARRES3* compared with shControl (Supplementary Fig. 2H).

### Suppression of RARRES3 attenuates the inhibitory effects of KDM2A depletion on malignant phenotypes of high-grade bladder cancer cells

**Fig. 5:**
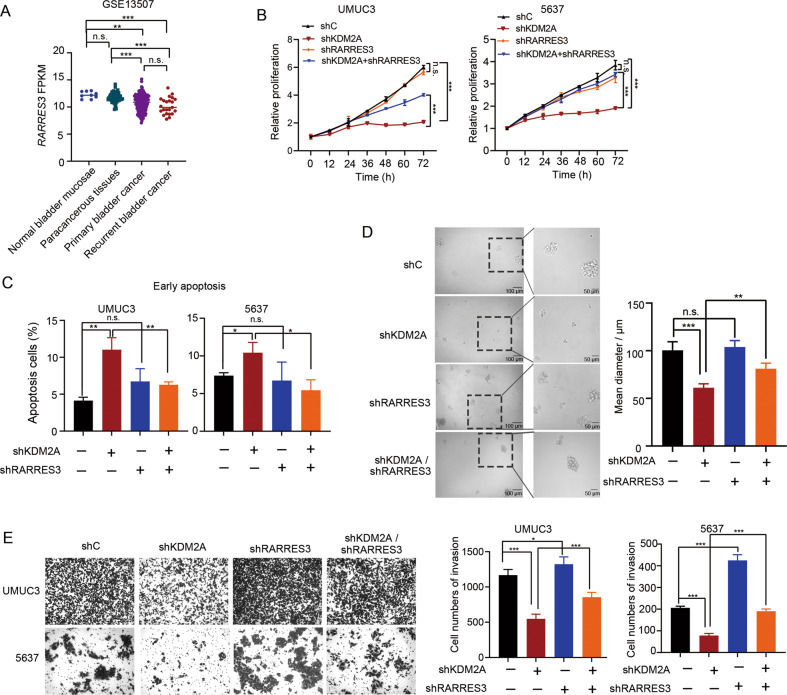
Suppression of RARRES3 contributed to the cell invasion function of KDM2A. **A** Analysis of RARRES3 expression level in bladder cancer tissues and normal bladder tissues in GEO database (GSE13507). **B** Cell viability of KDM2A KD cells with or without RARRES3 KD. **C** Early apoptosis analysis upon KDM2A KD and RARRES3 KD separately or simultaneously. **D** Sphere forming assay was performed on KDM2A KD UMUC3 cells with or without RARRES3 KD. The sphere size was measured at day seven. **E** Invasion assay was performed upon silencing KDM2A and RARRES3 separately or simultaneously. Cell invasion was assessed by counting the number of migrated cells after 24 h. *P < 0.05; **P < 0.01; ***P < 0.001 is based on the Student’s *t* test. All results are from more than three independent experiments. Values are mean ± SD.

By analyzing the Gene Expression Omnibus (GEO) dataset, we found that the expression of *RARRES3* in primary and recurrent bladder cancer was lower than that in the normal bladder mucosa and paracancerous tissues (Fig. 5A). To investigate the role of *RARRES3* in the phenotypes of bladder cancer cells regulated by KDM2A, we subcutaneously injected the shCtrl and shRARRES3 UMUC3 cells into nude mice. There was no significant difference in tumor growth between shCtrl and shRARRES3 groups (Supplementary Fig. 3A, B). Then, we generated KDM2A/RERRES3 double-KD UMUC3 and 5637 cells (Supplementary Fig. 3C). KDM2A silencing-induced cell growth inhibition was partially abrogated in the absence of *RARRES3* (Fig. 5B). In addition, a decrease in the proportion of early apoptotic cells was associated with *RARRES3* deficiency (Fig. 5C). Moreover, the sphere formation ability of *KDM2A* KD cells dramatically recovered after silencing *RARRES3* (Fig. 5D), and *KDM2A* depletion induced a decrease in invasive ability that was restored to the normal control by further *RARRES3* KD (Fig. 5E). We detected the expression of EMT related genes (Supplementary Fig. 3D) and KDM2A targeted genes (related to revised Supplementary Fig. 3E) in KDM2A/RARRES3 double mutants. The results indicate that the change of gene expression caused by KD of *KDM2A* can be partially restored by KD *RARRES3*.These findings suggested that KDM2A facilitates bladder cancer cell proliferation and invasion by inhibiting *RARRES3* expression.

### Combination therapy of IOX1 and ATRA synergistically suppresses proliferation of high-grade bladder cancer cells

**Fig. 6:**
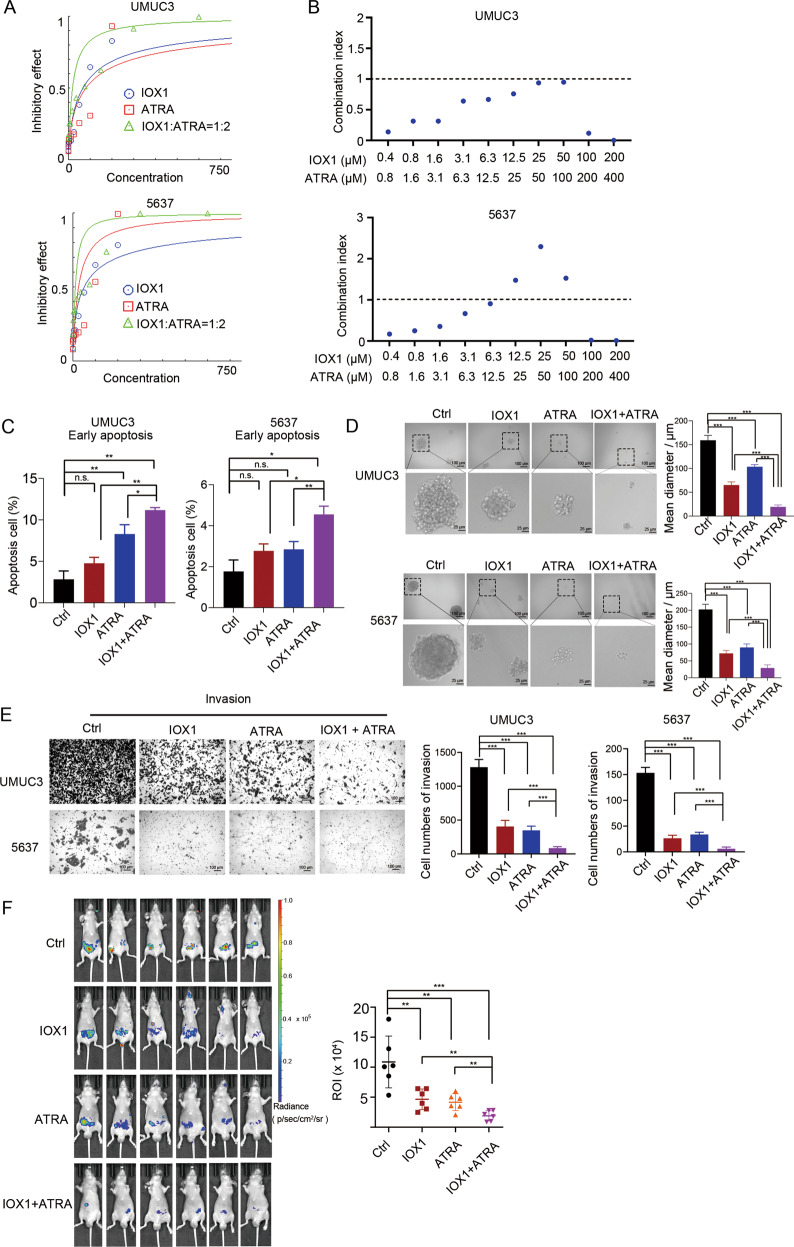
Combinatorial therapy with IOX1 and ATRA. **A** The concentration-effect curve showed the effect of IOX1 and ATRA combination at 1:2 ratio. **B** Combination index value in combinatorial drug treatments. UMUC3 and 5637 cells treated with IOX1 and ATRA in combination at indicated concentrations for 48 h. Cell viabilities were measured and normalized to DMSO control values. Combination index value (CI) of each drug combination condition was calculated using CalcuSyn software. CI < 1 indicated a synergistic effect between the two drugs. **C** Flow cytometry analysis of early apoptosis staining by annexin V and PI and cell cycle staining by PI in UMUC3 and 5637 cells treated with IOX1 and/or ATRA. **D** sphere formation assay of UMUC3 and 5637 cells treated with IOX1 and/or ATRA, the sphere size was measured on day seven. **E** Invasion assay of UMUC3 and 5637 cells treated with IOX1 and/or ATRA. Cell Invasion was assessed by counting the number of migrating cells after 24 h. **F** Bioluminescence in vivo imaging showed metastasis (*n* = 6 per group) of nude mice injection (i.v.) of luciferase-expressing UMUC3 cells. The IOX1 and/or ATRA were treated (i.p.) daily for 10 days. IOX1, 10 mg/kg, and ATRA, 20 mg/kg. *P < 0.05; **P < 0.01; ***P < 0.001 is based on the Student’s *t* test. All results are from more than three independent experiments. Values are mean ± SD.

Previous work has shown that the expression of RARRES3 could be upregulated by all-trans retinoic acid (ATRA) in several cancers [26, 27]. Consistently, ATRA increased *RARRES3* expression in bladder cancer cells (Supplementary Fig. 4A). However, the efficacy of the KDM2A inhibitors IOX1 and ATRA in the treatment of high-grade bladder cancer cells is limited (Supplementary Fig. 4B). As a result, we investigated whether cell proliferation could be disrupted by the combination of IOX1 and ATRA. Surprisingly, KDM2A inhibition, together with ATRA, synergistically reduced the viability of high-grade bladder cancer cells (Fig. 6A, B). Moreover, combination significantly promoted the cell early apoptosis (Fig. 6C) and suppressed sphere formation (Fig. 6D) in UMUC3 cells compared with IOX or ATRA treatment alone. The combination treatment significantly decreased the migration (Supplementary Fig. 4C) and invasion (Fig. 6E) of UMUC3 cells in vitro, compared with IOX or ATRA treatment alone. In addition, among all treatment groups, the combined treatment group is the most effective in inhibiting UMUC3 cell growth and metastasis in vivo (Supplementary Fig. 4D, Fig. 6F). Our findings support the notion that KDM2A blockade combined with ATRA treatment may be a promising therapeutic strategy for high-grade bladder cancer.

## Discussion

**Fig. 7:**
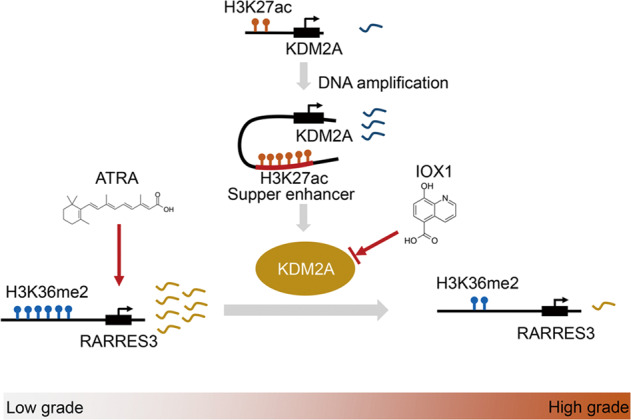
Schematic model of KDM2A regulation in human bladder cancer. Schematic model showed regulation of KDM2A in human bladder cancer. KDM2A was frequently amplified and implicated in SEs formation in high-grade bladder cancer. Frequent upregulation of KDM2A was attributed to decreased H3K36me2 enrichment at tumor suppressor RARRES3, thus downregulating mRNA levels. The combination of IOX1 (KDM2A inhibitor) and ATRA (RARRES3 activator) can reverse this regulatory process.

In summary, we provide novel evidence for the role of SE-driven KDM2A in modulating high-grade bladder cancer malignant phenotypes. In addition, we found that KDM2A inhibits RARRES3 expression by demethylating H3K36me2 at the *RARRES3* promoter. Targeting the KDM2A/RARRES3 axis may be a promising therapeutic strategy for high-grade bladder cancer (Fig. 7).

SEs are large enrichments of enhancers that bind high densities of transcriptional components, such as CBP/P300 and MED1, and histone modifications, such as H3K27ac and H3K4me1, to control prominent gene expression in cell identity determination. Tumor cells can form oncogenic SEs to create active binding sites for oncogenic driver genes via amplification [28, 29], DNA translocation [30, 31], and nucleation by small INDELs [32]. Our analysis showed that *KDM2A* copy number gain most likely contributes to the formation of SE at *KDM2A*, which leads to a high expression level of KDM2A in high-grade bladder cancer. Subsequently, *KDM2A* KD impaired bladder cancer cell proliferation, sphere formation, and migration. These results demonstrate that the SE-mediated increase in KDM2A expression resulted in the aggressive propensity of high-grade bladder cancer cells. Thus, suppression of SE may impair the aggressiveness of high-grade bladder cancer, which requires further investigation.

Our study also uncovered an important role of H3K36me2 in mediating high-grade bladder cancer phenotypes. The deposition, recognition, or removal of H3K36me2 is regulated by various epigenetic modifiers, such as NSD1 [33], NSD2 [34], NSD3 [35], ASH1L [36], SETMAR [37], and KDM2A [38]. As a matter of fact, KDM2A has been identified as a demethylase to demethylating H3K36me2, the covalent histone modification that involved in regulating a diverse range of biological processes including heterochromatin formation [39], X-chromosome inactivation and transcriptional regulation [40, 41], participating in regulating epithelial-mesenchymal identity, tumor differentiation, and metastasis [17]. H3K36me2 marks are critical for reinforcing the mesenchymal state by altering enhancer activity, as well as the corresponding promoter activity, and to regulate the transcription of key EMT regulatory factors [17]. Reduction in the demethylation of H3K36me2 enhanced the transcription of p21 and PUMA, thereby inhibiting the growth and metastasis of osteosarcoma [42]. Previous studies have demonstrated that serum and glucose deprivation induce KDM2A-mediated demethylation of H3K36me2 to decrease rRNA transcription and cell proliferation in breast cancer [13]. Our data support the notion that the inhibition of cell growth, sphere formation, and invasion induced by KDM2A silencing was partially abrogated in the absence of RARRES3 by an increase in H3K36me2 signal at the promoter of RARRES3. Our findings revealed that targeted restoration of H3K36me2 of *RARRES3* via KDM2A inhibition could restrain malignant progression in high-grade bladder cancer.

A series of studies have shown that RARRES3 is a tumor suppressor involved in cell growth and differentiation [43]. Loss of RARRES3 is considered a key driver of lung metastasis in estrogen receptor-negative (ER^-^) breast cancer, which disables cellular differentiation and engages in metastasis-initiating capabilities by facilitating adhesion of the tumor cells to the lung parenchyma [44]. In bladder cancer, RARRES3 expression was negatively correlated with KDM2A and was significantly downregulated. Also, RARRES3 was found to be abundantly expressed in well differentiated foci close to the bladder lumen, which represents the intermediate and umbrella cells of normal bladder urothelium. RARRES3 was identified as a retinoic acid responder, which is a regulator of gene transcription and an inducer of cellular differentiation that has long been associated with differentiation patterns in both normal and cancerous cells [27, 44]. We also found that ATRA can activate the expression of RARRES3 in bladder cancer but has little effect on bladder cancer cell growth. Interestingly, our data indicates that KDM2A inhibition combined with *RARRES3* activation can be a useful therapeutic strategy for high-grade bladder cancer. Future work is warranted to elucidate the mechanism of the synergistic effect in the control of cellular growth and metastasis in bladder cancer. Thus, we conducted the current research with the hypothesis that KDM2A-mediated H3K36me2 demethylation of *RARRES3* stimulates the progression of bladder cancer with its role in cell growth and metastasis.

## Supplementary information

Supplementary Information

checklist

Original Data File-western blot

## Data Availability

All data and materials used in the study are available in the manuscript.

## References

[1] Grayson M (2017). Bladder cancer. Nature.

[2] van Rhijn BWG, Burger M, Lotan Y, Solsona E, Stief CG, Sylvester RJ (2009). Recurrence and progression of disease in non-muscle-invasive bladder cancer: from epidemiology to treatment strategy. Eur Urol.

[3] Soukup V, Čapoun O, Cohen D, Hernández V, Babjuk M, Burger M (2017). Prognostic performance and reproducibility of the 1973 and 2004/2016 World Health Organization grading classification systems in non-muscle-invasive bladder cancer: a European Association of urology non-muscle invasive bladder cancer guidelines panel systematic review. Eur Urol.

[4] Knowles MA, Hurst CD (2015). Molecular biology of bladder cancer: new insights into pathogenesis and clinical diversity. Nat Rev Cancer.

[5] Babjuk M, Burger M, Compérat EM, Gontero P, Mostafid AH, Palou J (2019). European Association of urology guidelines on non-muscle-invasive bladder cancer (TaT1 and Carcinoma In Situ) - 2019 Update. Eur Urol.

[6] Network TCGAR. (2014). Comprehensive molecular characterization of urothelial bladder carcinoma. Nature.

[7] Wang L, Shilatifard A (2019). UTX mutations in human cancer. Cancer Cell.

[8] Audenet F, Isharwal S, Cha EK, Donoghue MTA, Drill EN, Ostrovnaya I (2019). Clonal relatedness and mutational differences between upper tract and bladder urothelial carcinoma. Clin Cancer Res.

[9] Dubuc AM, Remke M, Korshunov A, Northcott PA, Zhan SH, Mendez-Lago M (2013). Aberrant patterns of H3K4 and H3K27 histone lysine methylation occur across subgroups in medulloblastoma. Acta Neuropathol.

[10] Lu D-H, Yang J, Gao L-K, Min J, Tang J-M, Hu M (2019). Lysine demethylase 2A promotes the progression of ovarian cancer by regulating the PI3K pathway and reversing epithelial‑mesenchymal transition. Oncol Rep.

[11] Huang X, Pan J, Wang G, Huang T, Li C, Wang Y (2021). UNC5B-AS1 promotes the proliferation, migration and EMT of hepatocellular carcinoma cells via regulating miR-4306/KDM2A axis. Cell Cycle.

[12] Cao L-L, Du C, Liu H, Pei L, Qin L, Jia M, et al. Lysine-specific demethylase 2A expression is associated with cell growth and cyclin D1 expression in colorectal adenocarcinoma. Int J Biol Markers. 2018:1724600818764069. 10.1177/1724600818764069 29683067

[13] Tanaka Y, Yano H, Ogasawara S, Yoshioka S-I, Imamura H, Okamoto K (2015). Mild glucose starvation induces KDM2A-Mediated H3K36me2 Demethylation through AMPK To Reduce rRNA transcription and cell proliferation. Mol Cell Biol.

[14] Kong Y, Zou S, Yang F, Xu X, Bu W, Jia J (2016). RUNX3-mediated up-regulation of miR-29b suppresses the proliferation and migration of gastric cancer cells by targeting KDM2A. Cancer Lett.

[15] Liu L, Liu J, Lin Q (2021). Histone demethylase KDM2A: biological functions and clinical values (Review). Exp Ther Med.

[16] Kawakami E, Tokunaga A, Ozawa M, Sakamoto R, Yoshida N (2015). The histone demethylase Fbxl11/Kdm2a plays an essential role in embryonic development by repressing cell-cycle regulators. Mech Dev.

[17] Yuan S, Natesan R, Sanchez-Rivera FJ, Li J, Bhanu NV, Yamazoe T (2020). Global regulation of the histone mark H3K36me2 underlies epithelial plasticity and metastatic progression. Cancer Disco.

[18] Du J, Ma Y, Ma P, Wang S, Fan Z (2013). Demethylation of epiregulin gene by histone demethylase FBXL11 and BCL6 corepressor inhibits osteo/dentinogenic differentiation. Stem Cells.

[19] Wang Y, Zhu L, Guo M, Sun G, Zhou K, Pang W (2021). Histone methyltransferase WHSC1 inhibits colorectal cancer cell apoptosis via targeting anti-apoptotic BCL2. Cell Death Disco.

[20] Wagner KW, Alam H, Dhar SS, Giri U, Li N, Wei Y (2013). KDM2A promotes lung tumorigenesis by epigenetically enhancing ERK1/2 signaling. J Clin Invest.

[21] Ai Y, Wu S, Zou C, Wei H. Circular RNA circFOXO3 regulates KDM2A by targeting miR-214 to promote tumor growth and metastasis in oral squamous cell carcinoma. J Cell Mol Med. 2022;26:1842–52. 10.1111/jcmm.16533 PMC8918406 34117688

[22] Lin Q, Wu Z, Yue X, Yu X, Wang Z, Song X (2020). ZHX2 restricts hepatocellular carcinoma by suppressing stem cell-like traits through KDM2A-mediated H3K36 demethylation. EBioMedicine.

[23] Lu B, Zou C, Yang M, He Y, He J, Zhang C (2021). Pharmacological inhibition of core regulatory circuitry liquid-liquid phase separation suppresses metastasis and chemoresistance in osteosarcoma. Adv Sci (Weinh).

[24] Boeva V, Louis-Brennetot C, Peltier A, Durand S, Pierre-Eugène C, Raynal V (2017). Heterogeneity of neuroblastoma cell identity defined by transcriptional circuitries. Nat Genet.

[25] Bradner JE, Hnisz D, Young RA (2017). Transcriptional Addiction in. Cancer Cell.

[26] Zirn B, Samans B, Spangenberg C, Graf N, Eilers M, Gessler M (2005). All-trans retinoic acid treatment of Wilms tumor cells reverses expression of genes associated with high risk and relapse in vivo. Oncogene.

[27] Paroni G, Fratelli M, Gardini G, Bassano C, Flora M, Zanetti A (2012). Synergistic antitumor activity of lapatinib and retinoids on a novel subtype of breast cancer with coamplification of ERBB2 and RARA. Oncogene.

[28] Hnisz D, Abraham BJ, Lee TI, Lau A, Saint-André V, Sigova AA (2013). Super-enhancers in the control of cell identity and disease. Cell.

[29] Zhang X, Choi PS, Francis JM, Imielinski M, Watanabe H, Cherniack AD (2016). Identification of focally amplified lineage-specific super-enhancers in human epithelial cancers. Nat Genet.

[30] Gröschel S, Sanders MA, Hoogenboezem R, de Wit E, Bouwman BAM, Erpelinck C (2014). A single oncogenic enhancer rearrangement causes concomitant EVI1 and GATA2 deregulation in leukemia. Cell.

[31] Affer M, Chesi M, Chen W-DG, Keats JJ, Demchenko YN, Roschke AV (2014). Promiscuous MYC locus rearrangements hijack enhancers but mostly super-enhancers to dysregulate MYC expression in multiple myeloma. Leukemia.

[32] Mansour MR, Abraham BJ, Anders L, Berezovskaya A, Gutierrez A, Durbin AD (2014). Oncogene regulation. an oncogenic super-enhancer formed through somatic mutation of a noncoding intergenic element. Science.

[33] Fang Y, Tang Y, Zhang Y, Pan Y, Jia J, Sun Z (2021). The H3K36me2 methyltransferase NSD1 modulates H3K27ac at active enhancers to safeguard gene expression. Nucleic Acids Res.

[34] Popovic R, Martinez-Garcia E, Giannopoulou EG, Zhang Q, Zhang Q, Ezponda T (2014). Histone methyltransferase MMSET/NSD2 alters EZH2 binding and reprograms the myeloma epigenome through global and focal changes in H3K36 and H3K27 methylation. PLoS Genet.

[35] Yuan G, Flores NM, Hausmann S, Lofgren SM, Kharchenko V, Angulo-Ibanez M (2021). Elevated NSD3 histone methylation activity drives squamous cell lung cancer. Nature.

[36] Zhu L, Li Q, Wong SHK, Huang M, Klein BJ, Shen J (2016). ASH1L links histone H3 lysine 36 dimethylation to MLL Leukemia. Cancer Disco.

[37] Fnu S, Williamson EA, De Haro LP, Brenneman M, Wray J, Shaheen M (2011). Methylation of histone H3 lysine 36 enhances DNA repair by nonhomologous end-joining. Proc Natl Acad Sci USA.

[38] Chen L, Zhang J, Zou Y, Wang F, Li J, Sun F (2021). Kdm2a deficiency in macrophages enhances thermogenesis to protect mice against HFD-induced obesity by enhancing H3K36me2 at the Pparg locus. Cell Death Differ.

[39] Lachner M, O’Sullivan RJ, Jenuwein T (2003). An epigenetic road map for histone lysine methylation. J Cell Sci.

[40] Margueron R, Trojer P, Reinberg D (2005). The key to development: interpreting the histone code?. Curr Opin Genet Dev.

[41] Martin C, Zhang Y (2005). The diverse functions of histone lysine methylation. Nat Rev Mol Cell Biol.

[42] Wang Y, Sun B, Zhang Q, Dong H, Zhang J (2019). p300 Acetylates JHDM1A to inhibit osteosarcoma carcinogenesis. Artif Cells Nanomed Biotechnol.

[43] DiSepio D, Ghosn C, Eckert RL, Deucher A, Robinson N, Duvic M (1998). Identification and characterization of a retinoid-induced class II tumor suppressor/growth regulatory gene. Proc Natl Acad Sci USA.

[44] Morales M, Arenas EJ, Urosevic J, Guiu M, Fernández E, Planet E (2014). RARRES3 suppresses breast cancer lung metastasis by regulating adhesion and differentiation. EMBO Mol Med.

