# Supplementary material for: Histone H3K36me2 demethylase KDM2A promotes bladder cancer progression through epigenetically silencing RARRES3
Source: Cell Death Dis. 2022 Jun 13;13(6):547. doi: 10.1038/s41419-022-04983-7 (PMC9192503; doi:10.1038/s41419-022-04983-7)
Supplement: Supplementary file 1 — Supplementary Information [file 41419_2022_4983_MOESM1_ESM.docx]

**Supplementary Information**

**Histone H3K36me2 demethylase KDM2A promotes bladder cancer progression through epigenetically silencing RARRES3**

Bing Lu, Jiatian Wei, Houhong Zhou, Jie Chen, Yuqing Li, Liefu Ye, Wei Zhao, and Song Wu

**Contents**

**Supplementary Figures 1-4**

**Supplementary Materials and methods**

**
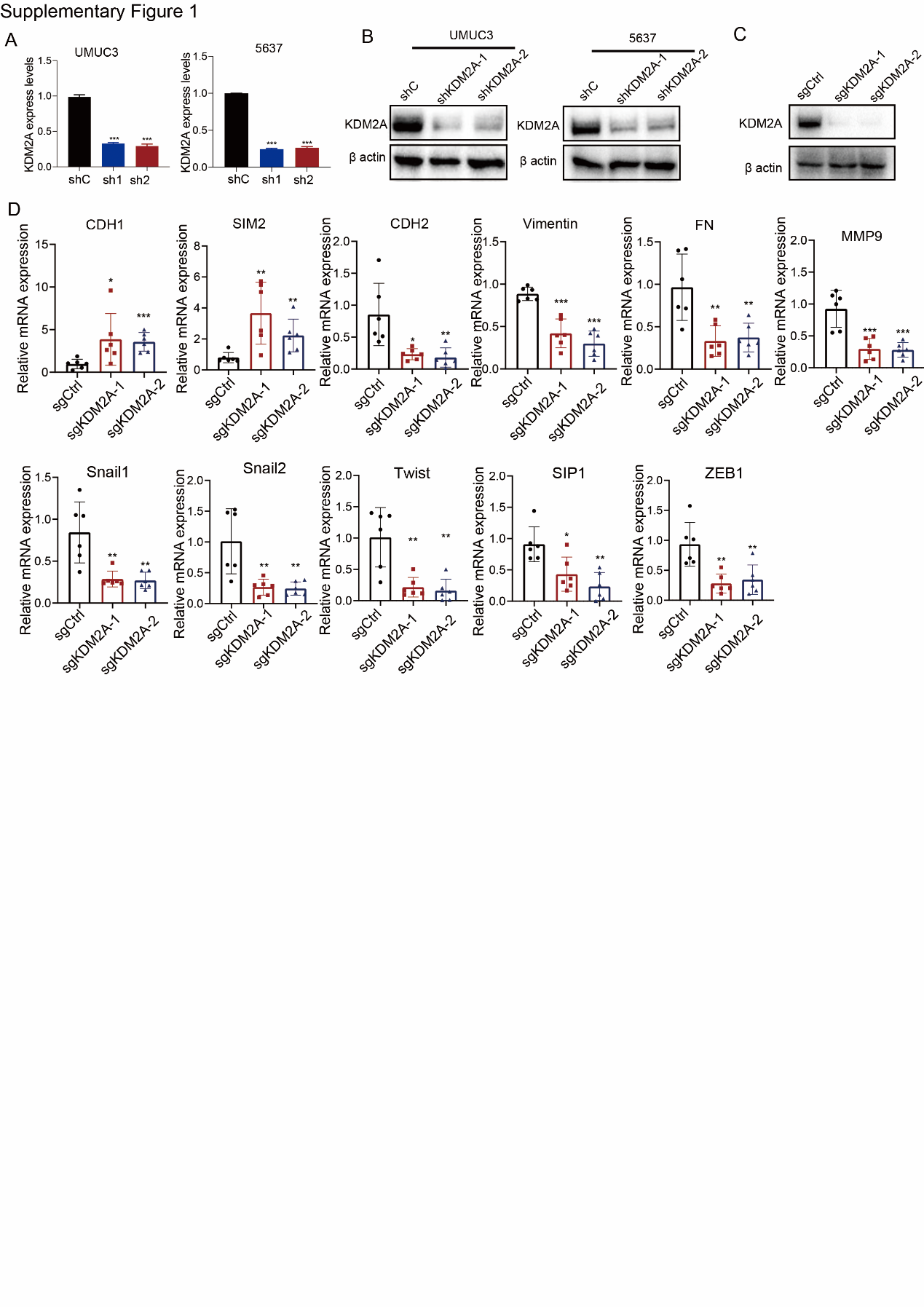
**

**Supplementary Figure 1.**

**A.** qPCR showed knockdown (KD) efficiency of KDM2A in UMUC3 and 5637 cells.

**B.** Verification of shKDM2A knockdown efficiency at protein level in UMUC3 and 5637 cells.

**C.** Verification of sgKDM2A knockout efficiency at protein level in UMUC3 cells.

**D.** The mRNA expression of MET/EMT-related genes in metastases by qPCR assays.

*, *P* < 0.05; **, *P* < 0.01; ***, *P* < 0.001 is based on the Student’s t test. All results are from more than three independent experiments. Values are mean ± SD.

**
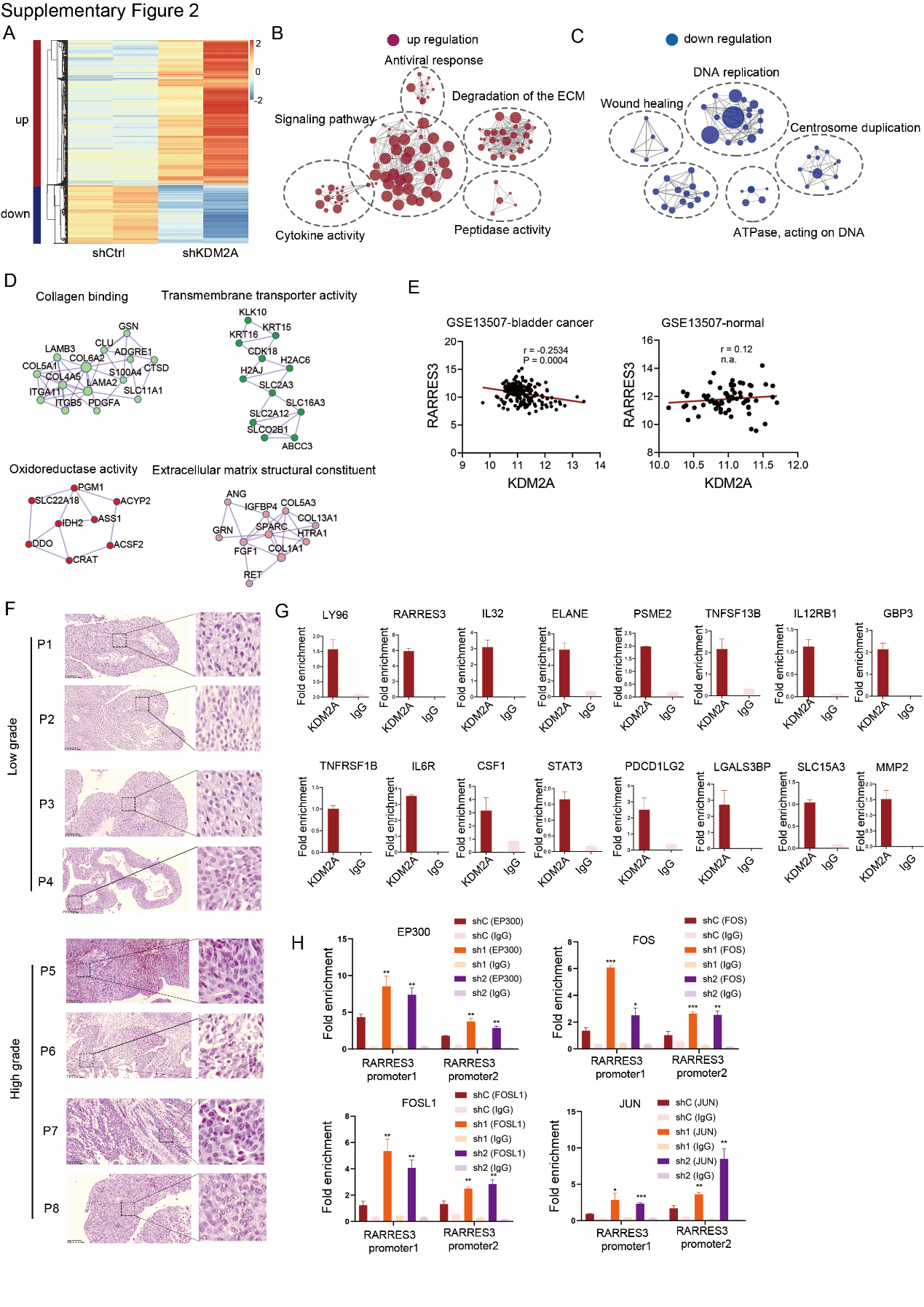
**

**Supplementary Figure 2. RARRES3 is a critical target of KDM2A.**

**A.** Heatmap showed the differential gene expression in shCtrl and shKDM2A UMUC3 cells.

**B, C.** Gene ontology (GO) analysis of the increased (B) and decreased (C) genes upon KD KDM2A.

**D.** GO terms of upregulated genes that KDM2A target directly.

**E.** Correlation analysis of KDM2A and RARRES3 expression in bladder cancer and normal tissue (GSE13507).

**F.** H&E staining of low-grade and high-grade bladder cancer tissue sections.

**G.** ChIP-qPCR showed the enrichment of KDM2A in its target gene promoter regions. **H.** ChIP-qPCR showed the transcriptional regulators binding on *RARRES3* promoter in UMUC3 cells with or without *KDM2A* shRNA transduction.

*, *P* < 0.05; **, *P* < 0.01; ***, *P* < 0.001 is based on the Student’s t test. All results are from more than three independent experiments. Values are mean ± SD.


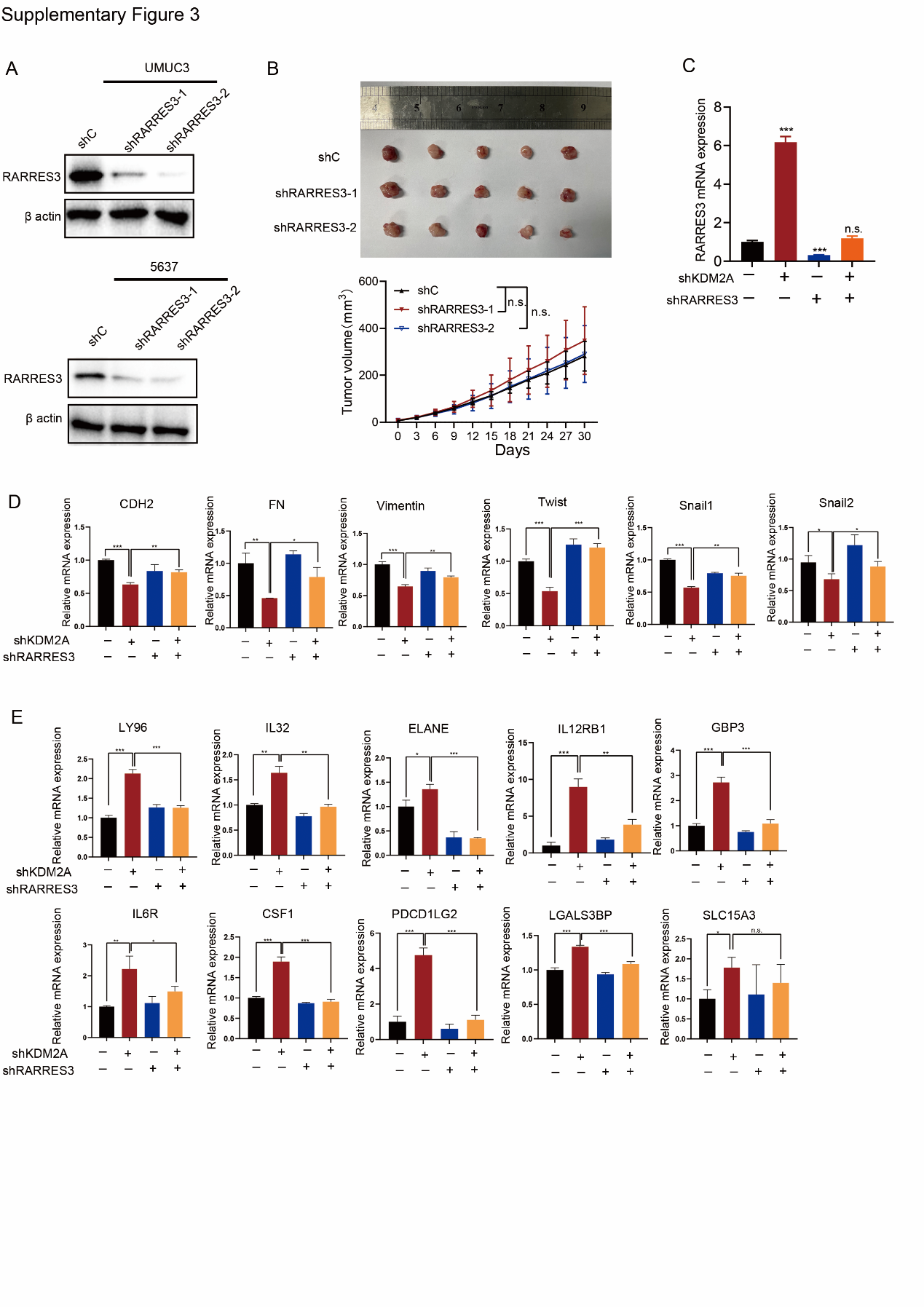


**Supplementary Figure 3**.

**A.** Verification of shRARRES3 knockdown efficiency at protein level in UMUC3 and 5637 cells.

**B.** Representative images and tumor growth curve of the xenograft from shCtrl and shRARRES3 groups.

**C.** qPCR showed the expression level of RARRES3 after KD KDM2A, RARRES3 separately or simultaneously.

**D.** The expression level of EMT related genes in UMUC3 cells transduced with indicated shRNA(s).

**E.** The expression level of KDM2A target genes in UMUC3 cells transduced with indicated shRNA(s).

*, *P* < 0.05; **, *P* < 0.01; ***, *P* < 0.001 is based on the Student’s t test. All results are from more than three independent experiments. Values are mean ± SD.


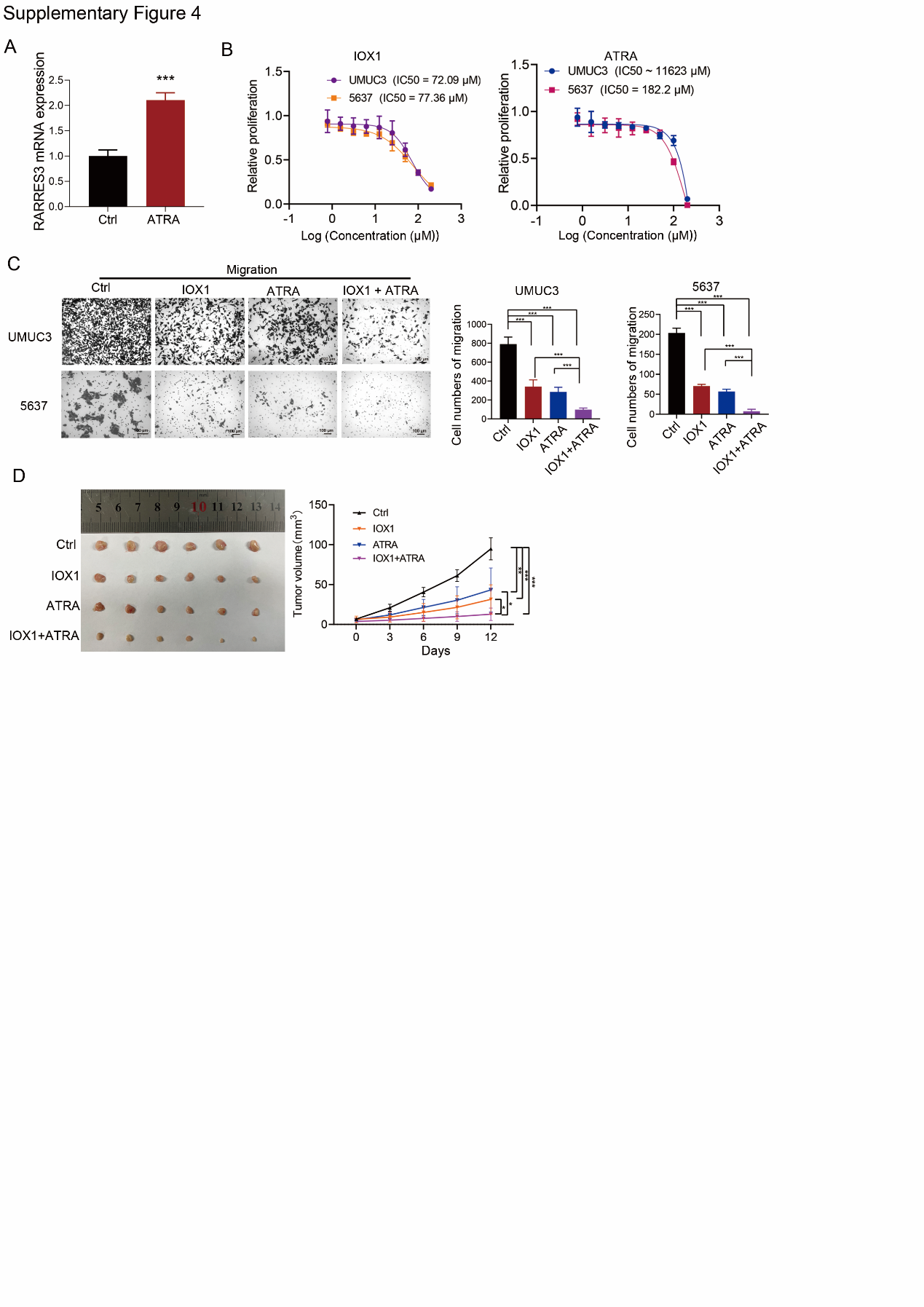


**Supplementary Figure 4. The effect of KDM2A inhibitor and RARRES3 agonist in bladder cancer cells.**

**A.** qPCR showed the expression level of RARRES3 with or without treat of ATRA for 48h in UMUC3 cells.

**B.** Cell viability of UMUC3 and 5637 cells treated with KDM2A inhibitor IOX1 (left) or RARRES3 activator ATRA (right) at indicated concentrations for 48h.

**C.** Migration assay of UMUC3 and 5637 cells treated with IOX1 and/or ATRA. Cell migration was assessed by counting the number of migrating cells after 24 h.

**D.** Representative images showing subcutaneous xenograft model in UMUC3 cells treated with IOX1 and/or ATRA (n = 6 per group). The IOX1 and/or ATRA were treated (i.p.) daily for 10 days. IOX1, 10 mg/kg, and ATRA, 20 mg/kg.

*, *P* < 0.05; **, *P* < 0.01; ***, *P* < 0.001 is based on the Student’s t test. All results are from more than three independent experiments. Values are mean ± SD.

**Supplementary Materials and methods**

**Western Blot**

Cells were lysed in RIPA buffer with protease inhibitor cocktail (Roche 4693132001). and were diluted with 0.25 volume to 5× SDS-PAGE Sample Buffer (GenStar). Gel electrophoresis was performed using SDS-PAGE, and proteins were transferred to PVDF Membrane (Bio-Rad). Membranes were incubated with following primary antibodies for 16–20 h at 4°C. Rabbit anti-KDM2A (Novus Biologicals, Cat. No. NB100-74602), Rabbit anti-RARRES3 (Proteintech, Cat. No. 12065-1-AP), Mouse anti-Beta actin (Proteintech, Cat. No. 60008-1-Ig).

**Immunofluorescence**

Bladder cancer tissue was prepared into paraffin sections. Paraffin sections were dewaxed, hydrated, antigen repaired, and followed by blocking in 10% BSA for 40 min at 37 °C. Tissue samples were incubated with KDM2A antibody (Novus Biologicals, Cat. No. NB100-74602), RARRES3 antibody (Proteintech, Cat. No. 12065-1-AP) and EpCAM antibody (Huabio, Cat. No. EM1111). After washes in PBS, cells were incubated with secondary antibodies (ABclonal, Goat anti-Rabbit , Cat. No. AS014, Goat anti-Mouse, Cat. No.AS003) in the dark. Cells were washed three times with PBS, 20μm ml^-1^ Hoechst 33258 (Life Technologies, H3569) was used to stain nuclei for 5 min at RT in the dark. After wash with PBS, Images were acquired at confocal microscopy.

**Real-time PCR primers used in this study**

| **Genes** | **Sequence (5’ to 3’)** |
| --- | --- |
| CDH1-forward | GGCCTGGACAGAGAGCAATG |
| CDH1-reverse | GGTTGACAAACCCTCTCCGT |
| SIM2-forward | ATCACTTCGCAGCTGGACAA |
| SIM2-reverse | ACGCGTCTCCTAAACCTTCG |
| CDH2-forward | GTCACCGTGGTCAAACCAAT |
| CDH2-reverse | TCCCTTGGCTAATGGCACTT |
| Vimentin-forward | AGGCGAGGAGAGCAGGATTT |
| Vimentin-reverse | AGTGGGTATCAACCAGAGGGA |
| Snail1-forward | CTATGCCGCGCTCTTTCCTC |
| Snail1-reverse | TGCTGGAAGGTAAACTCTGGATTA |
| Snail2-forward | TTAGAACTCACACGGGGGAGAAG |
| Snail2-reverse | TGTGCAGGAGAGACATTCTGG |
| FN-forward | GTGTGATCCCGTCGACCAA |
| FN-reverse | CACGGCCATAGCAGTAGCAC |
| Twist-forward | CTTCTCGGTCTGGAGGATGG |
| Twist-reverse | ATGACATCTAGGTCTCCGGC |
| MMP9-forward | TTCTGCCCGGACCAAGGATA |
| MMP9-reverse | ACATAGGGTACATGAGCGCC |
| ZEB1-forward | GTGACGCAGTCTGGGTGTAA |
| ZEB1-reverse | ATGGCTTCTCTCCACTGTGA |
| SIP1-forward | CGGAAACAAGGATTTCAGGGAG |
| SIP1-reverse | ACAGGAGTCGGAGTCTGTCA |
| LY96-forward | GAAGCAGTATTGGGTCTGCAA |
| LY96-reverse | TTGGAAGATTCATGGTGTTGACA |
| IL32-forward | TGGCGGCTTATTATGAGGAGC |
| IL32-reverse | CTCGGCACCGTAATCCATCTC |
| ELANE-forward | GGAGCCCATAACCTCTCGC |
| ELANE-reverse | GAGCAAGTTTACGGGGTCGT |
| PSME2-forward | AATCTTTTCCAGGAGGCTGAGG |
| PSME2-reverse | GGGAAGTCAAGTCAGCCACA |
| IL12RB1-forward | AGCTGCGTATGGAGTGGGA |
| IL12RB1-reverse | AGCTGCGTATGGAGTGGGA |
| TNFSF13B-forward | CCTCACGGTGGTGTCTTTCTA |
| TNFSF13B-reverse | AACGGCACGCTTATTTCTGCT |
| GBP3-forward | ATTCCCTGAAGCTAACGCAAG |
| GBP3-reverse | GGGCAGATCGAAGACAAAACATT |
| TNFRSF1B-forward | CCGGGAGCTCAGATTCTTCC |
| TNFRSF1B-reverse | GCTGCTACAGACGTTCACGA |
| IL6R-forward | CCCCTCAGCAATGTTGTTTGT |
| IL6R-reverse | CTCCGGGACTGCTAACTGG |
| CSF1-forward | TGGCGAGCAGGAGTATCAC |
| **Genes** | **Sequence (5’ to 3’)** |
| CSF1-reverse | AGGTCTCCATCTGACTGTCAAT |
| STAT3-forward | CAGGTTGCTGGTCAAATTCCC |
| STAT3-reverse | ACGTCCCCAGAGTCTTTGTC |
| PDCD1LG2-forward | ACCGTGAAAGAGCCACTTTG |
| PDCD1LG2-reverse | GCGACCCCATAGATGATTATGC |
| LGALS3BP-forward | AGGTACTTCTACTCCCGAAGGA |
| LGALS3BP-reverse | GGCCACTGCATAGGCATACA |
| SLC15A3-forward | TGGTGCCCTACTGGATGGT |
| SLC15A3-reverse | CTTCCGGGATCGTGTAGCTG |

| **Genes (promter)** | **Sequence (5’ to 3’)** |
| --- | --- |
| LY96-P-F | CTTTGCTCAGTGGAACCGTC |
| LY96-P-R | AACCACCCATGAACACCCTT |
| IL32-P-F | CCCTGCAGAGGGTCCTATCT |
| IL32-P-R | CAGGAACTGCCGGACCTAAG |
| ELANE-P-F | GGCCATTGTCTCCCTAACCC |
| ELANE-P-R | GGCTCTGACAATCTCTCCCG |
| PSME2-P-F | GGCCGCAAGCAGTAGGTAG |
| PSME2-P-R | GTCTCCGCGGTCCCACTTA |
| IL12RB1-P-F | CTGGAACCTAGGGAGGACTGA |
| IL12RB1-P-R | TTCCCATCACCTGCTGAGTTC |
| TNFSF13B-P-F | ACGACTCACCAGAAGAGGGA |
| TNFSF13B-P-R | GCCCCAGTCTAACAGGTACG |
| GBP3-P-F | GCTCAGTGCTGACTGCTTTAC |
| GBP3-P-R | CAGGAATCCCTGGGTTCAGAT |
| TNFRSF1B-P-F | GGGATAGGGAAACTGGCAGG |
| TNFRSF1B-P-R | GAGGGTGTGGCTGGTATGAC |
| IL6R-P-F | GTGGTTGCTCAAAAGCCAGG |
| IL6R-P-R | CCACCGTAAAGGCACAGTCT |
| CSF1-P-F | CCAGAGGTGACTGAGCATGG |
| CSF1-P-R | ATTCTCGGAGTGCCGAAAGG |
| STAT3-P-F | CTCTTACCACGCGGGAATCA |
| STAT3-P-R | GTGGTACGAGCGGTCTGAAT |
| PDCD1LG2-P-F | ATACCTGGGCCTTCCTTTGC |
| PDCD1LG2-P-R | TTCGTCCCATCGCTTGAACA |
| LGALS3BP-P-F | CTGGCAGCTTAGAGTCCGAG |
| LGALS3BP-P-R | CCGCACTTTCCCTACCATGT |
| SLC15A3-P-F | CGCCACGGGTAGAAAGAAGT |
| SLC15A3-P-R | CCGACTCACTTGTGGTCCTC |
| MMP2-P-F | CTGAAGTCAGGCGTTCCCAA |
| MMP2-P-R | TTGAATCCTTTCCTGCGCCA |
