# Supplementary figures and images for: Histone H3K36me2 demethylase KDM2A promotes bladder cancer progression through epigenetically silencing RARRES3
Source: Cell Death Dis. 2022 Jun 13;13(6):547. doi: 10.1038/s41419-022-04983-7 (PMC9192503; doi:10.1038/s41419-022-04983-7)

Supplementary Figure 1

B

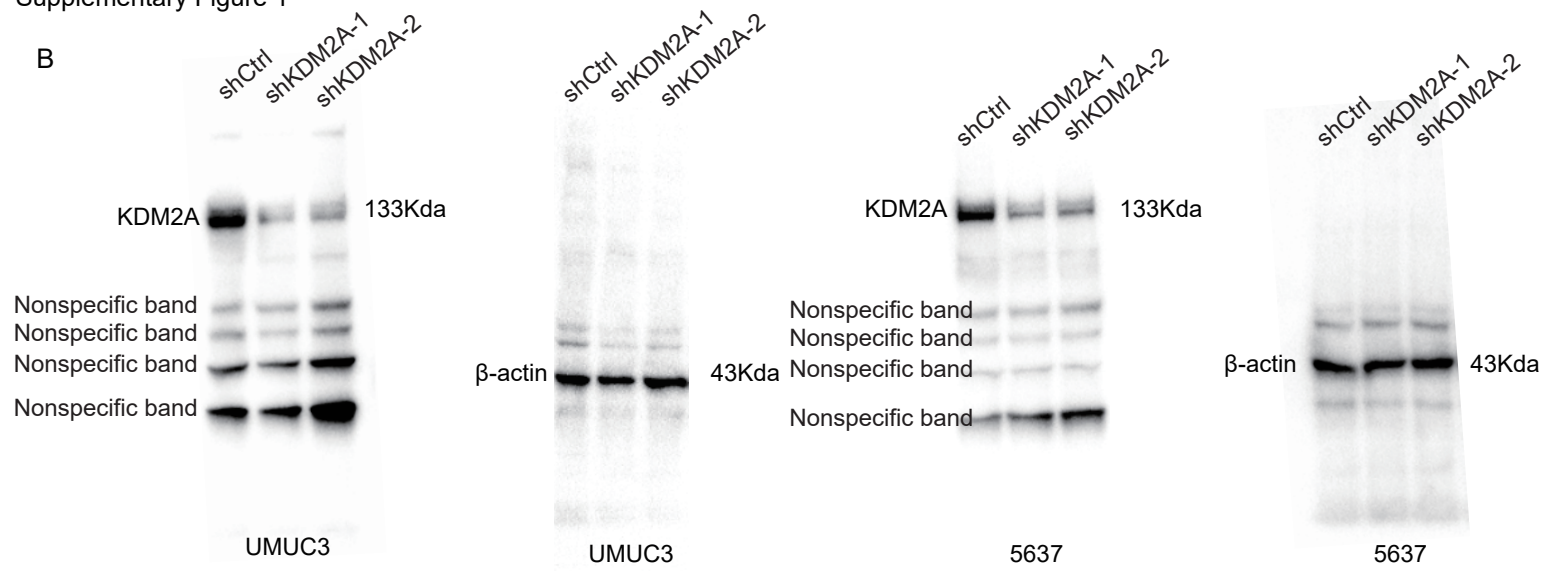

C

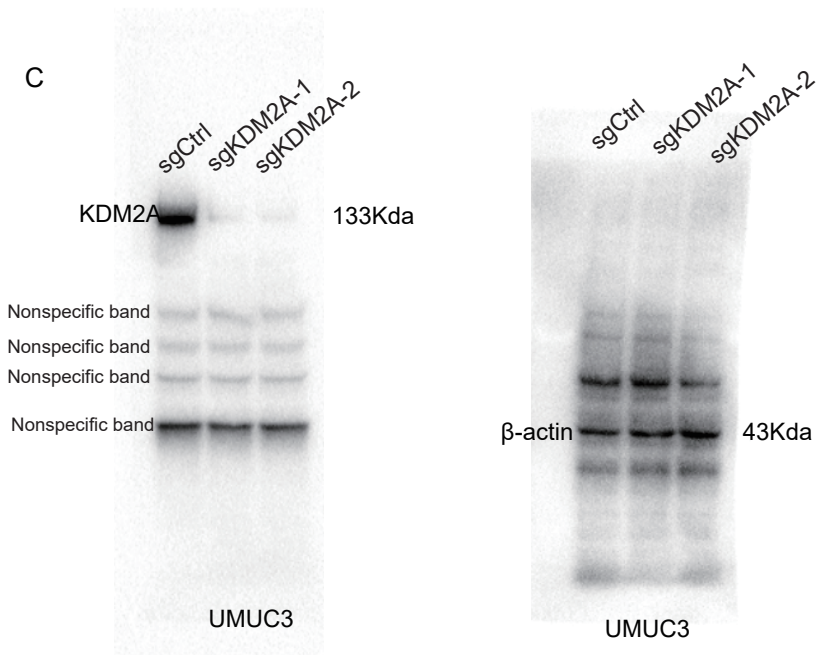

Supplementary Figure 3

A

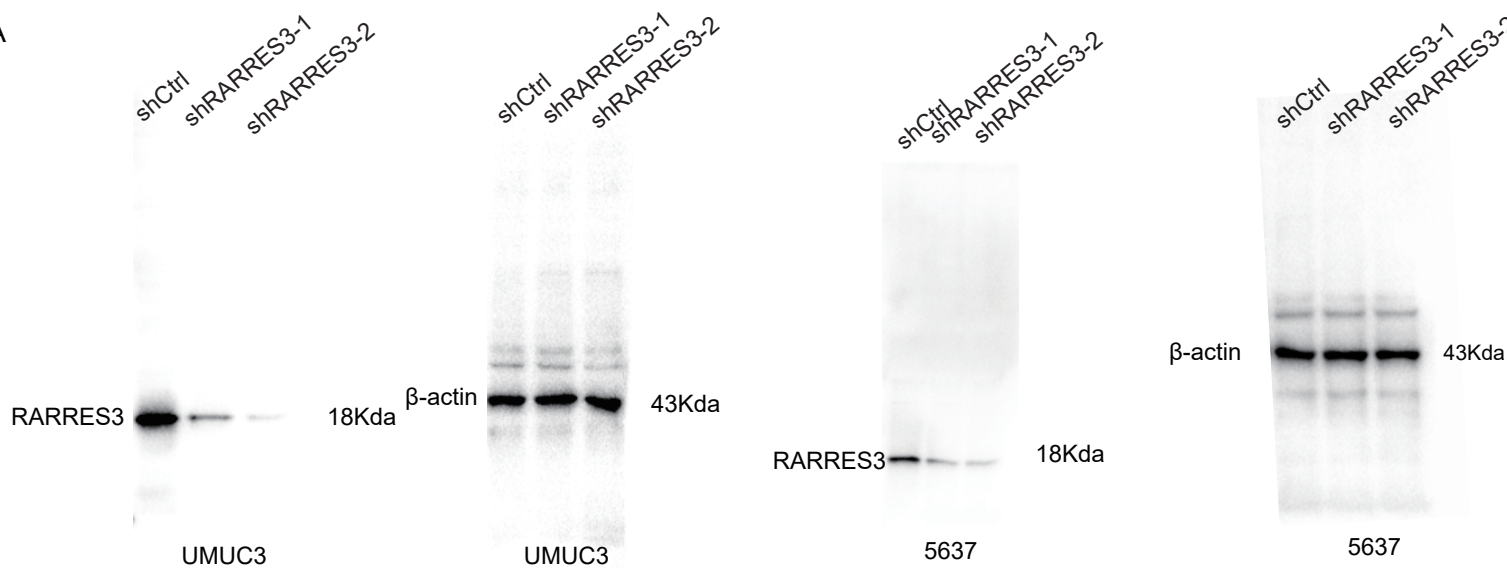

Supplement: Supplementary file 3 — Original Data File-western blot [file 41419_2022_4983_MOESM3_ESM.pdf]
